# Castor Bean, Cultivation

**Published:** 1874-12

**Authors:** 


The Cultivation of Castor Beans.—A California letter says of this crop: "The method of gathering and preparing for market is as follows: Every day the ripe spikes are gathered by hand, put in sacks, and hauled to the 'popping-ground,' which is a space of about an acre, made smooth and hard, like an old-fashioned buckwheat threshing ground. Here the spikes are spread, and during the day they pop open, from the heat of the sun, throwing out the beans. Each morning the straw is raked off, the beans shoveled up, cleaned in a fanning mill, and sacked, ready for market. By the time the field is once picked over, it is ready for another picking, like cotton, and the season, commencing in August, is not yet over. The yield is estimated at fifteen hundred pounds per acre, worth four cents per pound, or a gross yield of $60 per acre. The expense of cultivation, etc., is estimated this year at one-half this amount, but is greater than it probably will be another season, owing to inexperience and preparing new land. There is probably no crop so easily raised that will yield so large a return."

